# Therapeutics

**Published:** 1824-03-01

**Authors:** 


### Therapeutics.

1. *Injection of Opium into the Veins.*† From a great number of observations and reflections on the operation of poisonous substances, M. Coindet comes to the following conclusions.

1^mo.^ That there are but two classes of poisons—the first class comprehends the caustic and irritating substances—the second comprehends all the others.

2^ndo.^ That the poisons of the last class when taken into the stomach possess an intensity of action in an inverse ratio to their nutritive properties.

3^tio.^ That the decomposition of substances in the stomach varies with age, sex, health, and disease—in short, with the powers of digestion.

4^to.^ That the injection of medicinal substances into the veins may be very useful in certain diseases where the digestive function is so energetic that the said medicaments would be quickly decomposed in the stomach, and consequently prevented from entering the circulation unchanged, and acting on the system according to their peculiar properties.

The following remarkable case will illustrate these conclusions.

---

† Charles W. Coindet. Revue Medicale, Juillet 1823.

In December 1819, M. Coindet was called in consultation with M. Herey, to Jean Paterson, a young girl of 14 years, who was said to be labouring under violent tetanus. Three years previously, having been frightened by a dog, she fell into a nervous state, which soon assumed the form of well-marked hysteria. During four months the attacks took place every day at irregular hours. The catamenia now appeared, and the hysteric paroxysms ceased. The catamenia became obstructed, and the hysteria returned in the following manner. While sitting one day, at dinner, she was suddenly seized with rigid locking of the jaws, and in a few minutes afterwards became insensible. In two or three days the spasms extended from the head to the trunk, and assumed the appearance of real tetanus, still she remained insensible. In this state she had continued a week when our author first saw her. Various medicines were tried, but with little effect. The disease gradually ceased in about three weeks. In the middle of January 1820, M. Coindet was again called to her, and found the same symptoms as before described, but in a more aggravated form. The spasms were more violent than in the majority of cases of idiopathic tetanus. They commenced very irregularly in attacks of emprosthotonos—the head sometimes coming with violence against the knees. Opisthotonos succeeded; the body took the form of an arch, and rested on the heels and occiput. In these paroxysms, which sometimes lasted 20 minutes or more, the whole muscles of the body participated in the painful tension. The respiration was embarrassed—the pulsations of the heart became feeble and irregular—and the poor girl was threatened with suffocation! This horrible agony was broken up by some brisk convulsions, and succeeded by an interval of tranquillity. This succession of paroxysms had now lasted three weeks, and was increasing in degree when M. Coindet visited the patient. Her ordinary attendant had administered as much as an ounce of laudanum for a dose, without producing any effect on the system, which led our author to suppose that the remedies were decomposed in the stomach, and did not reach the circulation unchanged. It was therefore determined to inject a solution of opium into the veins. A scruple of opium was dissolved in distilled warmed water, and then filtered. There was a residue of 12 grains on the paper, but the narcotic principles were almost all in the solution. In the presence of several medical gentlemen, an opening was made into the vena basilica of the right arm, and the point of a fine syringe introduced, when a drachm was injected every five minutes, taking care not to allow any air to pass in, and to move the point of the syringe as little as possible in the vessel. At the *first* injection the respiration became more regular and less rapid—the state of the pulse and the other symptoms remaining the same. At the *second* injection the breathing became quite natural—the pulse rose to 100 and got fuller—the surface became a little elevated in temperature, and soon broke out in a gentle perspiration—the spasms were less violent—and the patient heaved some deep sighs, like a person awaking from sleep. At the *third* injection the pulse rose to 112, stronger and fuller—the skin became more coloured and covered with sweat—

the convulsions ceased almost entirely—and she articulated some words indistinctly. At the *fourth* injection the breathing became quicker, and the pulse rose to 120, very full, the skin being red, and the perspiration abundant. Several signs of returning sensibility occurred. At the *fifth* injection the foregoing phenomena were augmented in degree—the sight and hearing were entirely restored, the patient recognizing the physicians—she articulated distinctly some sentences, but like a person awaking out of a sleep troubled by dreams.

The operation was not followed by any alarming symptoms. The patient experienced some sickness at stomach in the night, with a little irregularity of breathing and precordial anxiety. The vein also shewed signs of inflammation, which were subdued by repeated applications of leeches and icy lotions in the track of the vessel. The patient described the effect of the injections as if a torrent of liquid fire had been poured through the veins of the arm, concentrating in her chest, and thence dispersed over the whole body, attended with violent heat and pricking of the skin. These sensations, she said, were of a most painful nature. She had a slight return of the spasms four days after the operation; but they readily yielded to the internal exhibition of opium. After an interval of complete health the disease again returned, but not in so violent a degree, as formerly. The use of the bath, and a discontinuance of strong irritating purgatives, which she was in the habit of taking, promptly restored her to health.

The foregoing experiment shews that opium may be injected into the vascular system, without always producing fatal effects; but even in the above case we conceive the young lady ran no small risk from the inflammation of the vein alone. Nevertheless, in desperate cases, as of hydrophobia, or tetanus, this *anceps remedium* may be worthy of a trial.

---

2. *Constipation of the Bowels.* On the 6th of May, 1822, a paper by Dr. W. Maxwell, was read at the Dumfries Medical Society, and subsequently published in the 78th number of the Edinburgh Medical Journal, on "*Constipation of the Bowels.*" Our author confines his observations to cases occurring without change of structure; dividing them into three sections. 1$^{mo.}$ constipation from indurated fæces—2$^{ndo.}$ from intus-susceptio and other visceral displacements—and *tertio*, from paralysis of the bowels. It is to the first class, almost exclusively, that Dr. M. directs his attention in the present paper, and endeavours to illustrate his subject by cases. The aim of the author appears to be to recommend two remedial agents of paramount power in this disease—namely, large injections of tepid linseed oil—and inflation by air, so as to distend the bowels and obviate the obstruction. In throwing up the oil, there should be a shoulder to the pipe to press on the anus, and prevent the return of the fluid during the operation. The quantity thrown up by Dr. M. is considerable—sometimes three or four pints. He mentions a case where he threw up 3½ gallons of warm water, before it reached the stomach—which, he thinks, it may always be made to do, notwithstanding the valve of the colon. "When a large portion of fluid is forced up by injection, the patient should be placed in a posture that will allow the bowels to hang at nearly right angles with the spine." What this posture is, our authar does not say—and we confess we are unable to divine. The practice of throwing up large quantities of fluid, so as mechanically to distend the bowels, is not new, as our readers know; but it is a measure which we think might be more frequently resorted to than it now is.

---

3. *Pharmaceutical Formulæ.** A correspondent in the London Medical and Physical Journal recommends a tincture of digitalis in spiritus ætheris nitrici (ʒj. to ℥iss.) in preference to the spirituous tincture now in use. If he can give no better reason for the change than the objection to the stimulus of the menstruum (in the present form) when exhibited in diseases of excitement, we think that objection "frivolous and vexatious." What possible effect can eight or ten drops of proof spirit have on the human frame taken three or four times a day? The idea is preposterous.

Mr. Sprague, an ingenious practitioner at Kingston, has offered a series of pharmaceutical observations on the pharmacopœia, in some late numbers of our respected cotemporary, from which we shall make a few extracts.

*Infusum Sarsaparillæ.* Mr. Brodie, we know, is in the habit of prescribing the following form of sarsaparilla, which is much less expensive than, and perhaps equally efficacious as, the simple or compound decoctions, now in use.

1. ℞. Sarsaparillæ radicis concisæ et contusæ ℥iss.

Glycyrrhizæ rad. contusæ . . . . ʒss.

Coriandri seminum contusæ . . . ʒij.

Liquoris potassæ . . . . . . . . . ʒi.

Aquæ ferventis . . . . . . . . . Oj.

Macera per horas xxiv. in vase leviter clauso et cola. A third part of this infusion may be taken thrice a day. Or the following:

2. ℞. Sarsæ radicis concis. et cont. . . . ℥iv.

Glycyrrhizæ rad. cont. . . . . . . ℥j.

Liquoris Calcis . . . . . . . . . Oiv.

Macera ut supra. A pint daily.

The following is the formula of vinum colchici introduced into Guy's Hospital by the late Dr. Marcet, and is considered by Mr. Sprague as the best method of preparing this important medicine.

3. "Take of the recent bulb of the colchicum (raised in *July* or *August*) sliced and bruised . 26 ounces.
Sherry wine . . . . . . . . . . . . 24 ounces.
Rectified spirit . . . . . . . . . . . 2 ounces.

Agitate the mixture twice a day, for seven days, and then filter for use."

---

* London Medical and Physical Journal, No. 298, and London Medical Repository, No. 120, 121.

There are numerous observations, original and collected, in Mr. Sprague's papers, which deserve the attention of the College, in the next edition of their Pharmacopœia, as well as of the professional public at large.

4. *Bodies in a State of Putrefaction.*—As the study of anatomy in this country is environed with so many difficulties, it becomes a great object, in every point of view, to preserve bodies, in our dissecting rooms, as much as possible in a state of freshness. M. Labarraque, in a memoir lately read before the Academy of Medicine, avers that a fluid composed of one pound of chloruret of lime in 30 or 40 quarts of water, when applied by means of a cloth dipped in it, to bodies in a state of putrefaction, will remove or prevent the fetor. He also proposes the substitution of liquid chloruret of lime for the acid gases commonly employed as disinfecting agents. This, we imagine, is not of very great consequence, as we believe that pure atmospheric air and ventilation are the best and surest disinfecters. Where we have fetid smells covered by acid gases, we are not apprised of the presence of the enemy.

P. S. Since writing the above, we observe a short paper in the *Journal Général de Medecine* which bears on the present subject.

M. Orfila was consulted by the police whether poison could be detected in a body that had been buried so long as 30 days? He answered in the affirmative, and was directed to exhumate a corpse and examine it in a medico-legal point of view. The body had been buried 32 days, and, when exposed to the open air, exhaled such a horrible fetor, that it could not be borne at two hundred yards' distance. After three hours' exposure, the fetor was little diminished, and it was determined to try the aspersion of solution of chloruret of lime. The mephitic exhalations ceased immediately; and Orfila and his assistants were enabled to prosecute the investigation for several hours without inconvenience. Arsenic was incontestibly detected in the intestines. In rendering homage to the merit of Labarraque's discovery, M. Orfila is of opinion that, by means of the article in question, we may proceed without danger or difficulty, to the examinations of bodies that have been several months interred.*

5. *Opium in Acute Mania.*† Mr. Allan, an intelligent surgeon in Leicester Square, London, has published a case illustrating the utility of large doses of opium in a paroxysm of acute mania. The patient had fallen down in a fit, but was found by Mr. A. talking incoherently, and with a dark brown tongue, and full firm pulse. He was bled to 32 ounces, and then had calomel and colocynth to open the bowels. Another epileptic fit occurred the succeeding day. Leeches—general bleeding—nauseating doses of antimony. No diminution of the mental alienation. Thirty-six leeches to the scalp,

* Journal Général de Medecine, August 1823.
† Mr. Allan. Ed. Journal, No. 77.

calomel and extract of rhubarb every four hours. Next day he was more outrageous than ever. Forty ounces of blood abstracted, which gave but temporary relief. Confined by a straight-waistcoat. Two grains of opium and ten of hyoscyamus statim, and to be repeated every four hours till sleep or quietude were procured. Passed a more noisy and restless night than ever. In the course of next day, however, he became quiet and fell into a state of stupor, from which he awoke refreshed, and soon regained possession of his mental faculties. His mouth was now affected by the calomel he had taken. He was a man addicted to drink, and the disease appears to have been closely allied to delirium tremens. It is probable that no extent of bleeding would have checked the disease had not opium been administered.

---

6. *Prussic Acid.** It is better than two years since Dr. Macleod published some observations on the good effects of prussic acid in some cases of dyspepsia. More extended experience has confirmed him in regarding this remedy as of considerable power in those forms of indigestion which are attended with much pain in the stomach and flatulence. Palpitation of the heart is not an unusual attendant on dyspepsia, sometimes simulating angina pectoris. In organic affections of the heart, too, we very generally have symptoms of dyspepsia, which aggravate the original disease. "In such instances of morbid action of the heart brought on by dyspepsia, or of dyspepsia sympathetic of organic disease of the heart, I am inclined, says Dr. Macleod, to think that much benefit is to be obtained from the employment of prussic acid."

---

*Leeches to internal Surfaces.* In the present volume of this Journal we have recorded the practice of Dr. Crampton respecting the application of leeches to internal surfaces affected with inflammation. It is but fair now to add, that three years ago, or more, Dr. Velpeau of Tours, presented to the public a memoir on the same subject, extracts from which are given in the Nouveau Journal de Medecine for July 1820. Dr. Velpeau observes, that he experienced the most salutary effects from the application of a single leech to the internal surface of the inferior palpebra, in cases of opthalmia. If the leech be applied about a line from the external border of the eyelid it will leave scarcely any trace of the bite, and produce no ecchymosis; but if it be allowed to fasten on the skin it will do both. The author confesses that he took the hint from Demours, who, in his treatise on diseases of the eye, recommends this practice.

P. S. We are informed by a respectable correspondent that Dr. Monro, of Edinburgh, was in the habit of applying leeches through the medium of a silver canula to inflamed tonsils, in severe cases of cynanche. He mentions this, not with the view of detracting from the merits of Dr. Crampton, but to insure to Dr. Monro the reputation of being the original inventor of the process.

---

* Dr. Macleod. Med. and Phys. Journal, No. 298.

8. *Obstruction of the Bowels.** Dr. Chisholm has related a case of obstinate constipation of the bowels relieved by *Read's injecting machine*, after various other means had failed. The obstruction had existed three or four days before Dr. Chisholm saw the patient with Mr. Beet, Surgeon, at Ashford. When seen by Dr. C. the patient's extremities were cold, and stercoraceous vomiting had come on. A tepid solution of yellow soap was prepared, and more than a wash-hand basin-full was gradually but perseveringly thrown up by means of the instrument abovementioned, and prevented from returning by napkins pressed to the anus. The patient's belly now resembled a drum. When the injection was allowed to come away, the spectators had the gratification to find it mixed with fæces. Shortly after this the patient passed flatus and stools, and all the bad symptoms quickly vanished. "*I have had many other cases, says Dr. Chisholm, where Read's machine was of infinite service, and I think every medical practitioner should have one in his possession.*" *Med. Repos. No.* 1, *New Series.*

9. *Arrest of Salivation.* Surgeon Sommé of Antwerp asserts that, mercurial salivation may be speedily arrested by the use of a gargle composed of one ounce of the superacetate of lead in two pounds of water. This gargle has the disadvantage of blackening the teeth, but is said to quickly heal those ulcerations of the mouth, which prove intractable under other means. In the ulcers of the tonsils and palate which occasionally follow mercurial courses, Mr. Sommé touches the parts with a hair pencil charged with the pure liquid of the acetate of lead.—*Archives Generales de Medecine.*

10. *Purpura Hæmorrhagica.*† It is well known that physicians are divided respecting both the pathology and treatment of this disease; some taking Dr. Parry's doctrine for their creed, and employing venesection, &c.; others viewing the disease as one of debility, and exhibiting the mineral acids, tonics, &c. Latterly some cases have been published where purgatives of oil of turpentine were found beneficial, and Mr. Thompson, of Whitehaven, has brought forward a case in support of this method of treatment.

It was a boy, five years old, whose body became covered with purple spots, accompanied by effusion of blood from different parts —pulse very quick—and other marks of pyrexia present. What was evacuated from the bowels was the same colour as that effused from the eyes and other parts. Turpentine was exhibited in small doses, sometimes combined with castor oil—and with a small allowance of port wine. Under this treatment, the boy gradually improved. The oil of turpentine was given by Mr. Thompson with the double view of acting on the bowels, and also of being absorbed into the system.

* Dr. Chisholm, Physician to the Canterbury Hospital.

† Mr. Ed. Thompson. Med. Repos. No. 119.

11. *Blood-letting in Hydrothorax.** Bleeding in dropsical affections is no new remedy ; but as hydrothorax is a formidable disease, and as there is a considerable antipathy against venesection existing among great numbers of the profession in a malady characterized by debility, we deem it proper to notice such cases as bear upon the subject in a practical point of view.

Mr. B. was called to a woman, 50 years of age, who was generally œdematous, with great oppression on the chest, inability to lie down —frequent cough with watery sputa—the sensation of a fluid in the chest when the body was changed—countenance œdematous and pallid—urine scanty, and not coagulable by heat—thirst—pulse 90, small and hard—disturbed sleep. After evacuating the bowels, it was determined to make a decided impression on the system, by drawing blood till syncope was produced. This required 36 ounces. She felt greatly relieved, and saline purgatives were continued, with supertartrate of potash in large doses. Two days afterwards, bleeding was again employed to syncope. The urine now increased in quantity—the pulse fell—the thoracic symptoms decreased. She rapidly recovered. The same practice was employed with success by the same writer in another case. Mr. Braid is deceived when he thinks that the presence of albumen in the urine, as shewn by its coagulating by heat, is considered as the *only* criterion for the utility of bleeding. Dr. Crampton and others have shewn that, little or no dependence is to be placed on this criterion.

---

12. *Supposed Hydrophobia.*† Great bruit was occasioned by this case at the Hotel Dieu, and re-echoed through Europe, by the Journal des Debats, It was, for some time, supposed that the monster hydrophobia had at last come under control, but this is not yet the case. The subject, however, is curious and interesting in a physiological point of view, and the particulars are worthy of record.

*Case.* Lazarus Beaufort, 25 years of age, a baker, was brought into the Hotel Dieu, on the night of the 14th October last. A disappointment in love had rendered him despondent for a fortnight previously, and led him into excesses in drink. Eight days before he entered the hospital, he had been seized with epistaxis, which continued three days without ceasing. The hæmorrhage stopped, he became delirious. On the 14th, in the afternoon, he was affected with strong convulsions. 15th (in the hospital) he was calm, but his speech was embarrassed—tongue red and rather dry. Presently he became greatly agitated and even convulsed, his face expressive of horror. He got furious, and attempted to bite every one around him, and spat in their faces, if presented drink or shewn any thing with a polished surface. Nevertheless he swallowed three or four times, though with difficulty, some drink which he called for. Generally he rejected liquids with horror. His pulse was very quick—skin

* Mr. Braid. Ed. Journal, No. 78.

† Archives Generales, Novembre, 1823.

pale, and covered with a viscid cold sweat—respiration interrupted. The patient had some lucid intervals, during which he answered distinctly to questions. Then again screaming, convulsions, &c. At this time a spot was discovered on one of the metacarpal bones of the right hand, of a reddish colour, depressed, circumscribed, and hard. It was supposed to have been the result of caustic. Some small scratches and traces of wounds were found on other fingers. His parents accounted for these by a fall which the patient had had on a broken vase; but M. Magendie thought the patient made some obscure allusion, in incoherent language, to the bite of a dog.* A large bleeding from the foot. At one P. M. Dr. Majendie returned and found the patient rather worse, and was convinced that the disease was hydrophobia. He determined to inject warm water into the veins. Into the radial vein of the fore-arm, Dr. M. injected about a pint and a half of tepid water, with facility, by means of a syringe, drawing off, at the same time, about six ounces of blood from the vein. Before the injection, the pulse was from 130 to 150. It gradually fell, and by the end of the operation, it came down to 80. The man called for drink, and swallowed it with ease. The convulsions nearly ceased—the countenance was calm though pale—he answered questions, and called frequently for drink—sweated profusely. At five, he asked to get up to make water, and passed about a pint of deep yellow urine. The pulse had got up again to 125—140 pulsations. 11 o'clock at night, patient was calm, sensible, and drank with ease. *16th October.* Slept three or four hours in the night. Was calm and sensible this morning, but had some difficulty in swallowing liquids. Pulse about 100, small and weak. Epigastrium tender to pressure—constipation—sense of tightness about the pharynx. In the course of the day a stool. Great debility—pulse 90. At 8 in the evening a paroxysm (of what, is not mentioned) increase of heat on the skin—frequency of pulse—slight subsultus tendinum. *17th Oct.* Three fetid stools in the night, almost entirely composed of blood—constant delirium—pulse now very small and depressed, about 90 in the minute. This morning cessation of the delirium—great thirst—liquids swallowed freely—some bouilli taken with appetite. In the course of the day, another bloody stool—alternately better and worse. In the evening another paroxysm. *18th.* Had a long and sound sleep in the night. This morning vomited some green matters—tongue dry—pulse more developed—the wound in the arm painful and tumefied—subsultus tendinum. *19th.* Vomiting this morning—tongue moist—thirst less urgent—black stools. *20th.* Vomiting—pallid countenance—small and frequent pulse—pain on pressure of the epigastrium—right lower extremity swelled, as were the wrist and elbow of the opposite side—great fear of death. *21st.* All the symptoms worse—much moral

* After the man's death, it was ascertained, that the above appearances were the result of a slight burn. The case was, therefore, *not* canine hydrophobia.

and physical prostration. 22*d.* Died at 2 o'clock this day, eight days after his admission into the hospital.

*Dissection.* We shall pass over many of the external appearances recorded by the authors, and which, we think, are quite insignificant. The vein operated on, and the surrounding cellular tissue were inflamed, above the wound. The brain slightly injected—some serous fluid in the ventricles—spinal arachnoid injected, and much sanguineous effusion in the cavity of the vertebral canal—mucous membrane of the bronchiæ red and injected throughout—the divisions of the bronchiæ filled with red and frothy mucus—some reddish serum in the pericardium—heart and veins in the neighbourhood filled with gas—substance of the heart pale—mucous membrane of the stomach offering two patches of inflammation—several ulcerations in the mucous membrane of the ileum near the cæcum—inner membrane of the colon inflamed in some places—a gazeous infiltration into the cellular membrane of the abdomen.

*Remark.* We have not yet read M. Magendie's observations on this curious case, but merely analyzed the statement as published in the Archives—the most authentic source of medical information in Paris. That the disease was *not* hydrophobia canina, no man of common information can for a moment doubt, notwithstanding the display made of it, *as such*, in certain non-descript medical newspapers in this country. One of these, pretending to wonderful celerity, published the case in *January* here, as an astonishing instance of early information, though it was published in Paris on the 1st of *November*, and the Journal containing it in the hands of most well-informed men in this country, more than six weeks! And, after all, the case was *not* hydrophobia. As a sequel to the curious case of Dr. Coindet's, we think it worthy of record, among the instances of dangerous and daring remedial agents.

---

13. *Strychnine and Brucine.** M. Andral has lately made many experiments with the strychnine, and also the brucine, (the alkaline salt of the angustura falsa,) from the results of which he is inclined to prefer the latter, as much less dangerous than the former. From his experiments on man and animals with both substances, he draws the following conclusions or corollaries:—$1^{mo.}$ Pure strychnine acts on man like the extract of nux vomica, but with infinitely greater intensity. $2^{ndo.}$ The action of strychnine is so powerful, that it should not be employed but with the greatest caution. $3^{tio.}$ Brucine may be commenced in doses of half a grain, and gradually increased. $4^{to.}$ Strychnine and brucine evince more or less effect on man, in a therapeutic point of view, according to the kind of paralysis for which it is administered. Thus, in paralysis connected with an inflammatory condition of the brain or spinal marrow, the above preparations aggravate the complaint; and so, in those cases of paralysis succeeding cerebral hæmorrhages, the employment of these alkalies is very

---

* M. Andral. Archives Generales, Octobre 1823.

useless if not dangerous. But there are other cases of paralysis where the complaint seems to continue after the absorption of the extravasated clot of blood; and here the alkalies may be very serviceable. Finally, these medicines are proper in cases where the paralysis does not depend on a lesion of the brain or spinal marrow, as in individuals who handle saturnine preparations.

These conclusions of our author are not to be implicitly relied on without much further trial; but the obstinate and deplorable disease against which these medicines have been administered lead the medical practitioner to investigate with care and attention every remedy that seems to offer a chance of success.

---

14. *Degeneration of Muscle.** M. Guersent lately presented to the Royal Academy of Medicine a case of what has been termed fatty degeneration of muscle. A child had been, from the age of three years, affected with rigid contraction of the right lower extremity—(permanent flexion of the leg on the thigh and of the thigh on the pelvis.) He died of croup. On dissection the spinal marrow was found healthy, as also the nerves issuing from it. The gluteus maximus was entirely destitute of its natural colour, and appeared like yellow wax, although the direction of its fibres could be easily traced and distinguished from the surrounding cellular tissue. The gastrocnemius muscle, in this little patient, was enormously developed, though remarkably pale, like those of other parts of the body.

